# Ternary Memristic Effect of Trilayer-Structured Graphene-Based Memory Devices

**DOI:** 10.3390/nano9040518

**Published:** 2019-04-02

**Authors:** Lei Li

**Affiliations:** 1 Key Laboratories of Senior-Education for Electronic Engineering, Heilongjiang University, Harbin 150080, China; lileidtk@hlju.edu.cn; Tel.: +86-13674621831; 2 Research Center for Fiber Optic Sensing Technology National Local Joint Engineering, Heilongjiang University, Harbin 150080, China

**Keywords:** GO@PBD/PMMA/GO@PBD heterostructure, ternary trilayer-structured memristor, graphene-based memory, tristable memory

## Abstract

A tristable memory device with a trilayer structure utilizes poly(methyl methacrylate) (PMMA) sandwiched between double-stacked novel nanocomposite films that consist of 2-(4-tert-butylphenyl)-5-(4-biphenylyl)-1,3,4-oxadiazole (PBD) doped with graphene oxide (GO). We successfully fabricated devices consisting of single and double GO@PBD nanocomposite films embedded in polymer layers. These devices had binary and ternary nonvolatile resistive switching behaviors, respectively. Binary memristic behaviors were observed for the device with a single GO@PBD nanocomposite film, while ternary behaviors were observed for the device with the double GO@PBD nanocomposite films. The heterostructure GO@PBD/PMMA/GO@PBD demonstrated ternary charge transport on the basis of *I*–*V* fitting curves and energy-band diagrams. Tristable memory properties could be enhanced by this novel trilayer structure. These results show that the novel graphene-based memory devices with trilayer structure can be applied to memristic devices. Charge trap materials with this innovative architecture for memristic devices offer a novel design scheme for multi-bit data storage.

## 1. Introduction

Memristic devices [1] have a remarkable potential for a complete renewal of electronic devices especially for memory, logic, and sensing applications. Binary memristic behaviors are commonly observed for the device with a single hybrid film. Research on multi-bit organic memory devices are considerably increasing for larger ultra-high-density data storage capacities of portable electronic devices. Multi-bit systems [2,3,4,5,6,7] provide an incremental data storage density without decreasing the size of a memory element. Based on previous studies on ternary storage [8,9,10,11,12], a common theory can be considered according to which a molecule containing two kinds of electron-withdrawing groups at different trap depths is a precondition for achieving ternary data storage. However, to what extent the two charge traps should differ to obtain ternary performance still remains unclear. In some studies, different electron-withdrawing groups were chosen blindly [13,14,15,16,17], leading to devices based on the designed molecule with two different electron-drawing groups that could not exhibit ternary memory behavior. Therefore, a rational way to build an innovative configuration to achieve a multilevel organic storage device is crucial. A multilayer structure permits improvement in dielectric properties [18], mechanical performance [19], thermal behavior [20], optical feature [21,22], and memory characteristics [23,24] of nanocomposite materials.

Inorganic/organic nanocomposites [18] doped with two-dimensional (2D) materials have been emerging as promising candidates for applications in next-generation nonvolatile memory devices, owing to light weight, flexibility, and printability. Two-dimensional nanomaterials have been showing a remarkable potential in the field of electronic devices [25], optoelectronic devices [26,27,28,29], van der Waals heterostructures [30], which present their outstanding physical, chemical, and optical properties. Graphene [31,32,33,34], an atom with a 2D layer of graphite, has prompted considerable research not only in materials science but also in condensed-matter physics. As the thinnest material composed of sp^2^-hybridized aromatic carbon atoms covalently packed into a continuous hexagonal lattice, graphene has a wide range of unique properties, for example, high three-dimensional aspect ratio and large specific surface area, superior area, superior mechanical stiffness and flexibility, remarkable optical transmittance, extraordinary thermal response, and excellent electronic transport properties. Various techniques have been investigated to manipulate the carbon atomic sheets in order to switch graphene-based nanocomposites between the ON-state and the OFF-state in nanoelectronic memory devices.

Tristable memory devices with trilayer structure utilize polymethyl methacrylate (PMMA) sandwiched between double novel nanocomposite films that consist of 2-(4-tert-butylphenyl)-5-(4-biphenylyl)-1,3,4-oxadiazole (PBD) doped with graphene oxide (GO). The heterostructure devices ITO/GO@PBD/PMMA/GO@PBD/Ni, which were fabricated by a solution-processable method, demonstrated ternary memristic effects. It was found that the tristable memory properties can be enhanced with a novel trilayer structure by varying the content of the charge traps formed between the small molecule and graphene. These results show that the novel graphene-based memory devices with a trilayer structure can be applied to memristic devices. We extensively characterized the surface morphology of the first and second GO@PBD nanocomposite layers by means of Scanning Electron microscopy (SEM). Both GO and its blend with PBD were characterized by transmission electron microscopy (TEM). The electrical properties of ITO/GO@PBD/PMMA/GO@PBD/Ni were implemented. To determine the memristic effect of graphene-base memory devices with novel trilayer-structure, memory devices with a single GO@PBD nanocomposite layer were examined as well. The carrier transport and the memory mechanisms of the devices were described on the basis of *I*–*V* fitting curves and energy band diagrams. The novel trilayer-structured ternary memristic memory device provided further enhancement of the device switching performance, which is discussed. 

## 2. Materials and Methods

The insulating polymer PMMA (molecular weight *M*_w_ = 120,000–150,000) was purchased from ARKEMA, with a purity of 99.5%. Commercially available glass substrates coated with indium tin oxide (ITO, 10 Ω/sq) were employed after cleaning with acetone, methanol, ethanol solvents, and distilled water. GO@PBD nanocomposites (GO and PBD powder were separately purchased from Hengqiu Tech. Inc., Suzhou, China and Sigma, Saint Louis, MO, USA) were prepared by mixing a solution of GO in 1-methyl-2-pyrrolidone (NMP) and PBD at a weight ratio of 10:1 and solution concentration of 3.5 mg/mL. Ultrasonic agitation was used to mix the solution for 4 h at room temperature. To prepare a PMMA (ARKEMA, Shanghai, China) solution, 1 mg of PMMA was added to 10 mL of chlorobenzene, followed by sonication for 4 h. The fabrication of the memory device was begun with spin coating of a GO@PBD nanocomposite layer on top of the cleaned ITO substrate. After a wetting time of 10 s, spin coating was performed at 1000 rpm for 60 s. This process was repeated for the second GO@PBD nanocomposite layer after the PMMA layer was transferred on the first GO@PBD nanocomposite film at 3000 rpm for 60 s to deposit the insulating layer. After spin coating, all films were baked in vacuum at 80 °C for 1 h. As top electrodes, 250 nm thick circular Ni electrodes with a diameter of 200 μm were deposited by a shadow mask with the diameter of 0.2 mm. The as-fabricated devices were kept overnight at ambient temperature. 

We extensively characterized the surface morphology of the first and second GO@PBD nanocomposite layers by means of SEM (Themoscientific, Waltham, MA, USA) up to 40,000× magnification, with working distance of 4.6 mm and high voltage of 20,000 kV. Both GO and its blend with PBD were characterized by a JEM-2100 TEM (JOEL, Tokyo, Japan) with the accelerating voltage of 200 kV. A NanoMap 500LS 3D Profilometer (aep Technology, Santa Clara, CA, USA) was used to measure the film thickness. TGA (TA Instruments, New Castle, DE, USA) as well as derivative thermogravimetry (DTG) were used to analyze the thermal properties of PBD and GO@PBD composite under a nitrogen atmosphere at a heating rate of 10 °C/min. The nitrogen flow rate for the balance and the samples was kept constant at 50 mL/min. All thermal experiments were conducted in the temperature range of 40–600 °C with at 10 °C/min heating rate. UV–visible absorption spectra of PBD, GO, GO@PBD blend, and PMMA solutions were obtained with a U3010 UV–vis spectrophotometer (HITACHI, Tokyo, Japan) in the range of 200–500 nm. Fluorescence spectra were measured with a F-4500 FL Spectrophotometer (HITACHI, Japan), in 2.5 nm/2.5 nm slit widths and at scan speed of 1200 nm/min. The electrical properties of ITO/GO@PBD/PMMA/GO@PBD/Ni were implemented by a semiconductor device analyzer (Keithley 4200SCS; Keithley, Solon, OH, USA). To determine the memristic effect of graphene-base memory devices with novel trilayer structure, memory devices with a single GO@PBD nanocomposite layer were examined as well.

## 3. Results

**Figure 1:**
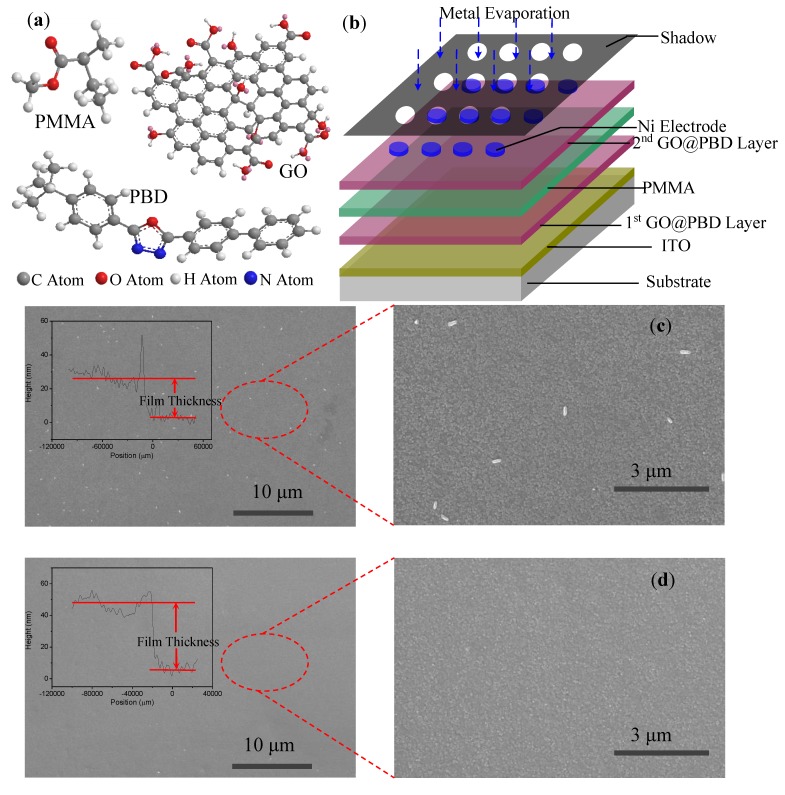
(**a**) Chemical structures of the molecules poly(methyl methacrylate) (PMMA), 2-(4-tert-butylphenyl)-5-(4-biphenylyl)-1,3,4-oxadiazole (PBD), and graphene oxide (GO); (**b**) schematic diagram of the device’s structure; (**c**,**d**) morphological characteristics of the surfaces foofr the first and second GO@PBD nanocomposite layers. Inset of (**c**,**d**): thickness of the first nanocomposite film and trilayer structure.

The chemical structures of PMMA, PBD, and GO are shown in Figure 1a. We introduced PBD, a small molecule which has an oxidiazole moiety and electron-transfer properties, for possible use in electronic memory devices. GO has chemically reactive oxygen functionalities, including carboxylic acid groups at the edges and epoxy and hydroxyl groups on the basal planes, and is an electrically insulating material due to the disrupted sp^2^ bonding networks. As shown in Figure 1b, the heterostructure consisted of an insulating polymer PMMA sandwiched between double PBD embedded in GO nanocomposite layers. The nanocomposite film, which could have charge-trapping sites and support the small molecules rather than the polymer backbone of polymer memory devices, may have memristic properties for nonvolatile memory applications. SEM images revealed finely dispersed morphologies after the first and second GO@PBD nanocomposite layers were spin-coated on the ITO substrate, as shown in in Figure 1c,d. A profilometer was used to measure the thicknesses of the fist GO@PBD nanocomposite film and the trilayer structure, which were estimated to be 20 nm and 45 nm, respectively. We fabricated devices consisting of single or double GO@PBD hybrid films stacked in a PMMA layer. These devices had binary and ternary nonvolatile memristic behaviors, respectively. The morphology of the surface of the first GO@PBD nanocomposite layer presented some “white dots” that stemmed from the organic small molecule PBD.

**Figure 2:**
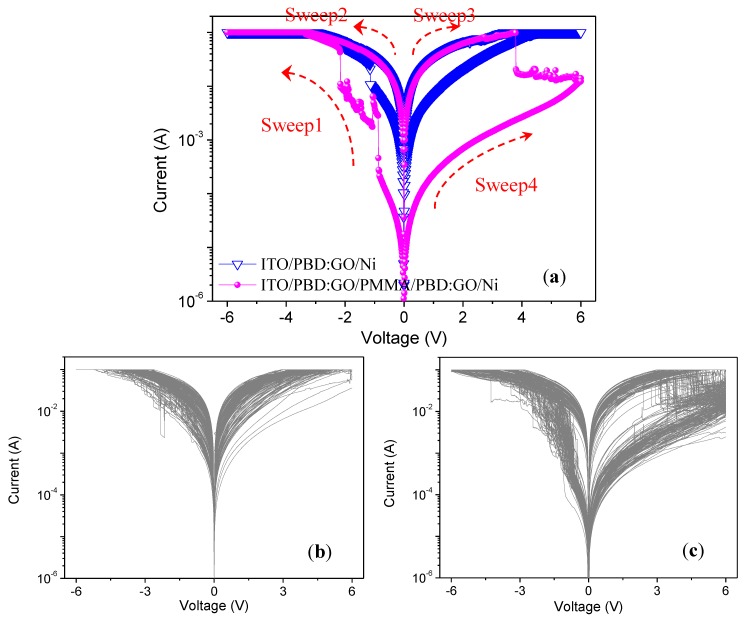
(**a**) *I*–*V* characteristics of tin oxide (ITO)/GO@PBD/Ni and ITO/GO@PBD/PMMA/GO@PBD/Ni. Reproducibility of (**b**) binary and (**c**) ternary memristic behaviors as shown by 100 consecutive *I*–*V* curves.

Figure 2a presents the typical *I*–*V* characteristics of a GO@PBD film between an ITO bottom electrode on a glass substrate and a Ni top electrode (ITO/GO@PBD/Ni). This curve was initially recorded by scanning the applied voltage from 0 to −6 V (Sweep1) and then sweeping once again (Sweep2), followed by a positive scan from 0 V to 6 V (Sweep3 and Sweep4) with limited current compliance up to 10^−1^ A. When the bias voltage was applied to the Ni top electrode (from 0 V to −6 V), a transition from the OFF-state (namely, high-resistance state, HRS) to the ON-state (namely, low-resistance state, LRS) occurred, presumably because of the oxidation of 1,3,4-oxadiazole groups. As the negative bias voltage increased, the current started in the OFF-state and abruptly grew to reach the ON-state at a threshold voltage of −1.14 V. The conductive ON-state was kept even in the reverse sweep range from 0 V to 3.27 V. Above 3.27 V, the device sharply switched to the OFF-state. The *I*–*V* characteristics of GO@PBD were binary, symmetric, dipolar, stable, and reproducible. Next, we characterized the device ITO/GO@PBD/PMMA/GO@PBD/Ni. Figure 2a shows the representative *I*−*V* curves of the device ITO/GO@PBD/PMMA/GO@PBD as well. Unlike the device ITO/GO@PBD/Ni, this device exhibited ternary memristic behaviors. When the applied voltage was swept from 0 to −6 V, two abrupt electrical transitions occurred. One transition was from HRS to an intermediate resistance state (IRS) at *V*_SET1_ = −0.87, and the other transition was from IRS to LRS at *V*_SET2_ = −2.16 V. Subsequently, only one transition from LRS to IRS was observed at *V*_RESET_ = 3.79 V when the applied voltage was swept to positive voltages, and then the current declined from IRS to HRS when the applied voltage grew from 3.80 V to 6 V. Furthermore, when the applied voltage was gradually swept to positive voltages from 0 V to 6 V, the device reached a HRS. These results suggested that the first transition might have occurred when the injected charges were trapped in the first nanocomposite film, and the second transition occurred when they were trapped in the second one. The *I*−*V* curves suggested that the GO@PBD layer acted as a trap and detrapped layers and that resistive switching was caused by charges trapped in the nanocomposite films. In addition, binary memristic behaviors were observed for the device with a single GO@PBD layer, and ternary memristic behaviors were observed for the device with the trilayer structure GO@PBD/PMMA/GO@PBD. These results demonstrated that devices consisting of a single PMMA layer sandwiched between double-stacked GO@PBD hybrid films can be applied to multi-bit nonvolatile memristic devices. The reproducibility of the binary and ternary memristic behaviors with 100 consecutive *I*–*V* characteristics is shown in Figure 2b,c.

**Figure 3:**
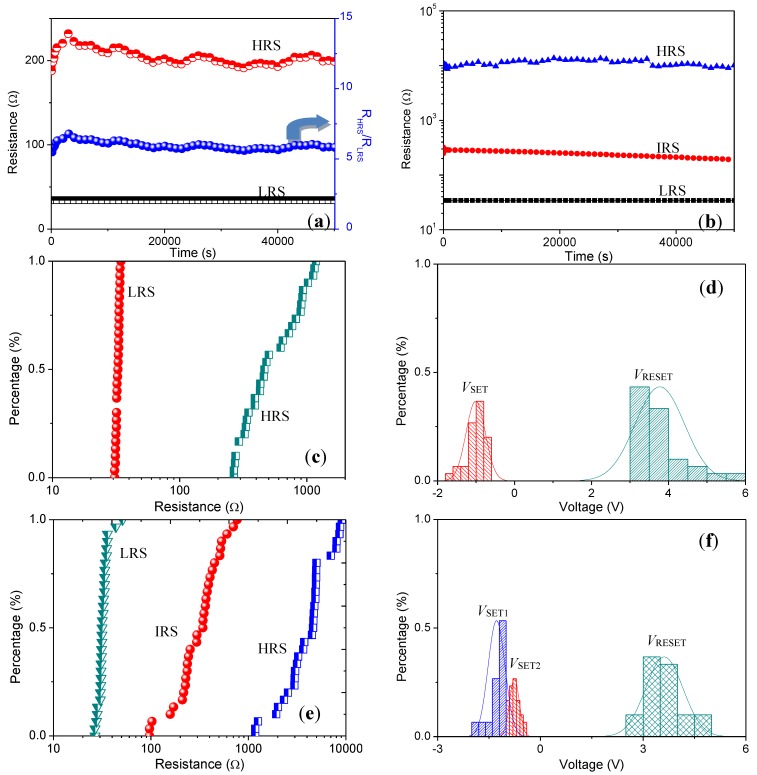
Retention of (**a**) ITO/GO@PBD/Ni and (**b**) ITO/GO@PBD/PMMA/GO@PBD/Ni; Device-to-device profiles of ITO/GO@PBD/Ni for (**c**) the resistance in LRS (*R*_LRS_) and HRS (*R*_HRS_), (**d**) the bias switching from HRS to LRS (*V*_SET_) and from LRS to HRS (*V*_RESET_); Device-to-device profiles of ITO/GO@PBD/PMMA/GO@PBD/Ni for (**e**) the resistance in LRS (*R*_LRS_), in IRS (*R*_IRS_), and in HRS (*R*_OFF_), and (**f**) the bias transiting from HRS to IRS (*V*_SET1_), from IRS to LRS (*V*_SET2_), and from LRS to IRS (*V*_RESET_).

We performed a retention time test on the devices ITO/GO@PBD/Al and ITO/GO@PBD/PMMA/GO@PBD/Ni (Figure 3a,b). At a constant read voltage of −0.1 V, the ON and OFF states was maintained for longer than 5 × 10^4^ s. With the help of GO, the device showed no significant degradation under ambient atmosphere. As a result, new organic memory devices could provide a stable retention time and multiple switching cycles. A correlative property in a memory cell is its ability to retain information for long periods of time. For device uniformity, the data distribution of 30 samples separately for ITO/GO@PBD/Al and ITO/GO@PBD/PMMA/GO@PBD/Ni devices was examined, as demonstrated in Figure 3c–f. The cumulative distribution of *R*_LRS_ and *R*_HRS_ at a read voltage of −0.1 V, *V*_SET_, and *V*_RESET_ is displayed in Figure 3c,d, where the means (standard deviation) of *R*_LRS_ and *R*_HRS_ are 33 (1) Ω and 596 (298) Ω, respectively. The statistical analysis of ITO/GO@PBD/PMMA/GO@PBD/Ni indicated in Figure 3e,f illustrates that the means (standard deviation) of *R*_LRS_ (at a read voltage of −1 V), *R*_IRS_ (at a read voltage of −1 V), and *R*_HRS_ (at a read voltage of −0.1 V) are 33 (5) Ω, 347 (165) Ω, and 4.5 (2.3) kΩ, respectively. Therefore, the resistive switching ITO/GO@PBD/Al, similarly to ITO/GO@PBD/PMMA/GO@PBD/Ni, is electrically bistable. Nevertheless, the trilayer-structured memory device has a smaller threshold voltage range and an *I*_LRS_/*I*_IRS_/*I*_HRS_ current ratio close to 1:10^2^:10^3^.

**Figure 4:**
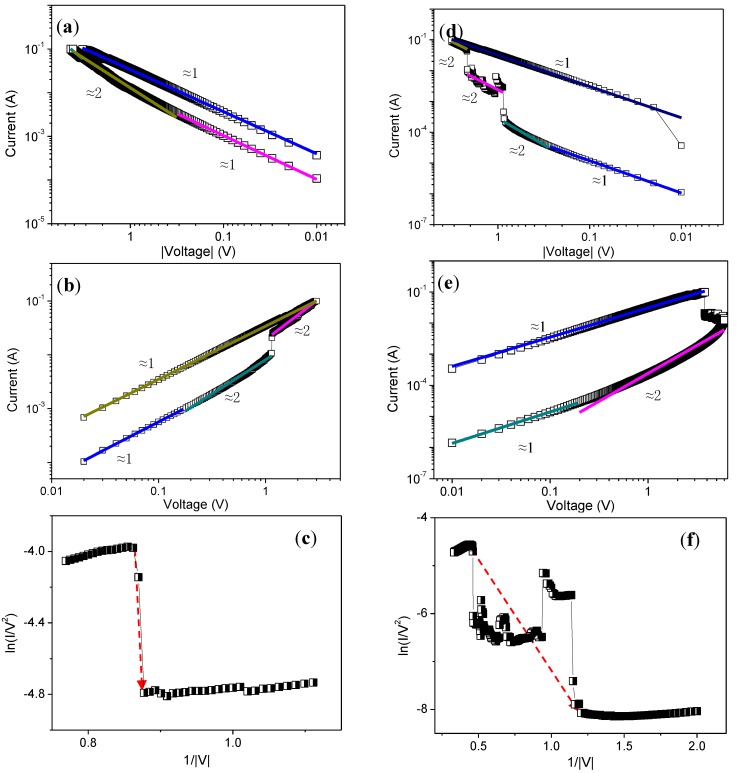
Fitted *I*–*V* curves of ITO/GO@PBD/Ni in (**a**) negative and (**b**) positive sweeps in low-resistance state (LRS) and high-resistance state (HRS); Fitted *I*–*V* curves of ITO/GO@PBD/PMMA/GO@PBD/Ni scanning from (**d**) negative and (**e**) positive sweeps; F–N tunneling mechanism for (**c**) ITO/GO@PBD/Ni and (**f)** ITO/GO@PBD/PMMA/GO@PBD/Ni during the set process.

Further theoretical and experimental analyses were performed to clarify the detailed ternary memristic effect. To take into account carrier injection processes for ITO/GO@PBD/Ni, the *I*−*V* curves were plotted in log−log scales in Figure 4a,c. At low bias voltages, the current increased linearly with the voltage, consistent with Ohmic law. Nevertheless, for −1.14 V < *V* < −0.17 V, the current increased according to the relationship *I* ∝ *V*^2^ in terms of a trapped-charge-limited current (TCLC) model. The fitted curves for LRS corresponded to the Ohmic law. When the voltage was applied to the ITO electrode, charges were injected to the lowest unoccupied molecular orbital (LUMO) level of the PBD layer and were transported in the direction of the applied voltage through the tunneling among the PBD molecules (*V* < *V*_SET_) [10], as demonstrated in Figure 4c. Because PBD acts as an electron-acceptor and GO@PBD can be seen as an electron-transporting layer, the injected charges were trapped in the GO@PBD nanocomposite layer. Consequently, an internal electric field was induced in the PBD (*V* ≈ *V*_SET_). For *V* < *V*_RESET_, some of the injected charges were free and were accelerated by the local internal field. This LRS was maintained until the charges trapped in the GO@PBD nanocomposite layer were released by applying positive voltages (*V* ≈ *V*_RESET_). As a result, the device switched to HRS (*V* > *V*_RESET_). 

To examine the ternary memristic effect of the trilayer structure, the *I–V* curves were divided into three regimes, exhibited in Figure 4d,e. When the device was in the HRS region, the slope of the fitted *I*–*V* plot seemed to transit from 1 to 2, indicating the dominance of space-charge-limited conduction (SCLC) before setting. Moreover, SCLC played an important role in the IRS region during the second turn-on of the resistive switching. The *I*–*V* graph in this region was re-plotted, as shown in Figure 4f. There was an increase in the current value with multiple ON states, with a negative slope. The observed behavior might be consistent with Fowler–Nordheim (F-N) tunneling, as suggested by the negative slope of the straight red lines in the ln(*I*/*V*^2^)-versus-1/*V* graph. When the device reached LRS after switching, Ohmic conduction occurred, and the *I*–*V* plot showed a linear behavior of the device based on a slope of ~1.

**Figure 5:**
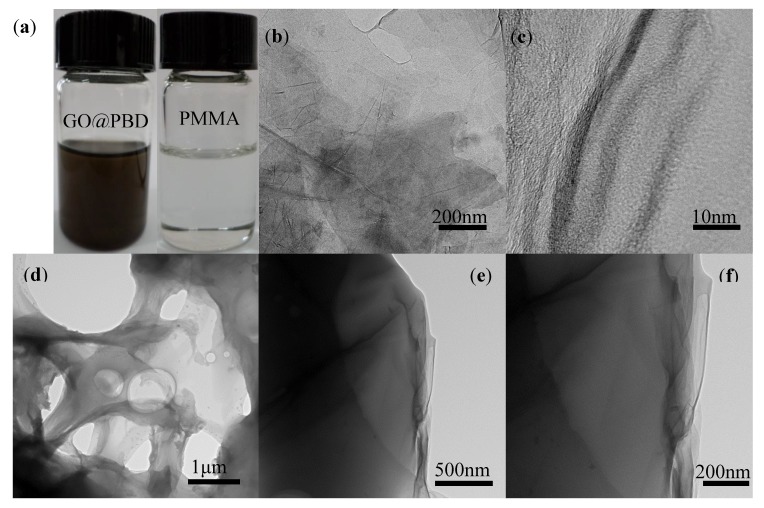
(**a**) Photos of GO@PBD and PMMA solutions; (**b**) TEM and (**c**) high-resolution transmission microscopy (HRTEM) images of GO at 20,000× and 40,000× magnification; (**d**–**f**) PBD and GO in the chemical ratio 10:1 at 5000×–40,000× magnification.

The GO surface exhibited a wrinkled and aggregated morphology, as shown in the TEM images of Figure 5a. As indicated in Figure 5b–e, the TEM images obtained from GO sheets and GO@PBD with the chemical ratio of 10:1 revealed that GO had roughly three layers. This suggested that the hydrogen bonding interaction led to a homogeneous dispersion of the GO sheets in the composites. However, some folding cluster aggregations were observed in the composite with a high GO content, due to the strong π–π stacking within GOs. Furthermore, a smooth surface structure of the heterostructure spin-casted on the ITO-substrate was obtained, indicating that GO was well dispersed in PBD.

**Figure 6:**
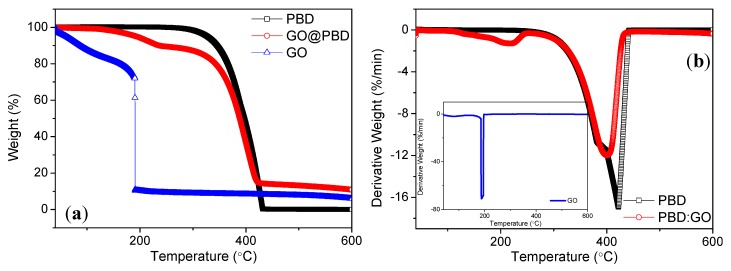
(**a**) TGA and (**b**) DTG properties of PBD, GO@PBD nanocomposite, and GO.

For further investigation of the ternary memristic effect of the trilayer-structured graphene-based memory device, the thermal stability of the samples PBD, GO@PBD, and GO was also analyzed under a N_2_ atmosphere at a scanning rate of 10 °C /min by TGA. The TGA curves of PBD, GO@PBD composite, and GO are presented in Figure 6a. It is clear that the initial thermal decomposition temperature of pure PBD samples was much higher than that of the GO@PBD blend. There is a very interesting observation that must be noted. Two steps in the degradation were observed for the GO@PBD composite, although the degradation of PBD proceeded in a single step. Concerning Table 1, the onset temperature (temperature at 10% mass loss, *T*_0.1_) decreased by 109 °C for GO@PBD compared to virgin PBD. In contrast with virgin PBD, the mid-point temperature (temperature at 50% mass loss, *T*_0.5_) decreased by 9 °C for the GO@PBD composite. For TGA and DTA of GO, a gradual weight loss was observed in the temperature range from 40 °C to 170 °C, which was assigned to the removal of residual or absorbed water molecules. At 188 °C, abrupt weight loss was observed with a strong exothermic peak, which was assigned to the decomposition of oxygen containing functional groups. As shown in Figure 6b, the GO@PBD nanocomposite showed two intensive weight loss steps between 40 °C and 260 °C and between 260 °C and 440 °C, respectively, while PBD had only one degradation step between 300 °C and 400 °C. The DTG curves of the GO@PBD nanocomposite exhibited strong peaks at 400 °C, whereas PBD displayed strong peaks at 423 °C. This suggests that GO incorporation into PBD had an inhibiting influence on the thermo-oxidative degradation of PBD.

**Figure 7:**
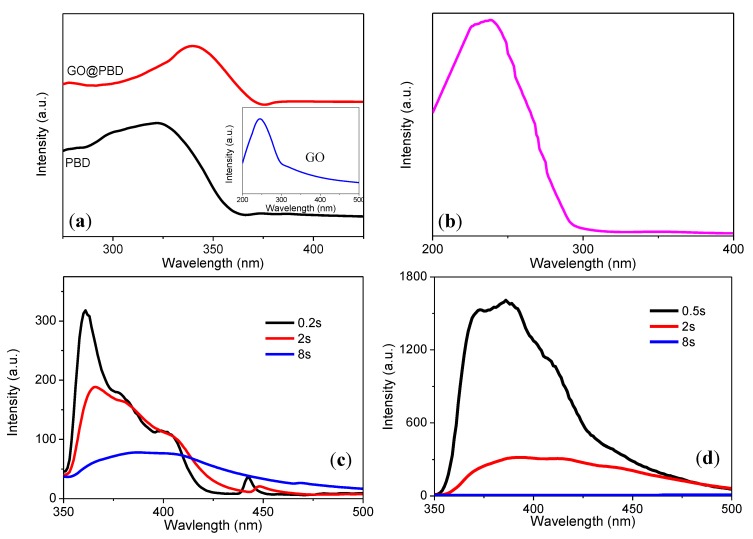
(**a**) UV–vis absorption spectra of PBD, GO@PBD, and GO in 1-methyl-2-pyrrolidone (NMP) solution; (**b**) UV–vis absorption spectra of PMMA. Fluorescence (**c**) excitation and (**d**) emission spectra with various response times.

The UV–visible absorption spectra of PBD and its composite in solution were characterized. The absorption maximum of PBD at 322 nm was assigned to 1,3,4-oxadiazole, as indicated in Figure 7a together with that of GO in the inset. The typical absorption peak at 245 nm could be assigned to the π − π* transition of the graphitic sp^2^ domains. The energy band gap (*E*_g_) could be estimated as the onset energy of the absorbance spectra, from the following equation:

$$E_{g}=h\times c/\lambda_{edge}$$

where *h* is the Planck constant, *c* is the light velocity, and *λ*_edge_ is the linear approximation of the absorption edge to the wavelength axis. Table 2 shows the *E*_g_ values of PBD, GO@PBD, GO, and PMMA in solution. This simple method allowed estimating and then comparing the energy gaps to investigate the composite in solution.

To further understand the tristable trilayer structure, fluorescent spectra for PBD in terms of distinct response time were measured by monitoring the excitation at 440 nm and emission at 360 nm for the response times 0.2 s, 2 s, and 8 s. Figure 7c,d shows the fluorescence excitation and emission spectra, respectively, dependent on response time at room temperature. The maximum excitation and emission appeared in a red shift when increasing the response time. The experimental results indicated that the fluorescence could be affected by the response time. The carriers excited from an isolated light-emitting center at the surface of nanocrystallites can tunnel into the crystallite core. Because many surface states may exist together on the surface of PBD, which will result in different decay time constants, it is reasonable to assume that the two slower decay processes could be attributed to the different surface states that could be regarded as trap sites for next-generation nonvolatile memory. The fluorescence spectra showed a systematic red shift with an increase in response time, and this self-quench phenomenon could result from the formation of excimers or exciplexes in a highly concentrated solution [35].

**Figure 8:**
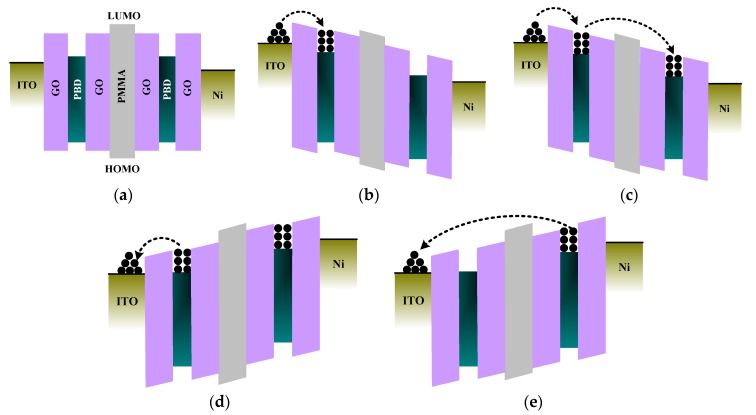
Schematic diagrams of the trilayer structure corresponding to the conduction mechanisms of the device: (**a**) initial state; (**b**) HRS→intermediate resistance state (IRS); (**c**) IRS→LRS; (**d**) LRS→IRS; (**e**) IRS→HRS.

The energy-band diagrams for the memory mechanisms of ITO/GO@PBD/PMMA/GO@PBD/Ni are shown in Figure 8. The values of *E*_g_ between the LUMO and the highest occupied molecular orbital (HOMO) for GO, PBD, and PMMA were obtained from the results in Table 2. The wide *E*_g_ of the PMMA layer suggests that it can be seen as a charge-blocking layer. As for the GO@PBD layer, the difference between the energy levels of GO and PBD led to the formation of potential wells as electron trapping and detrapping centers, as GO has a wider energy gap (4.143 eV) than PBD (3.324 eV). Therefore, the GO@PBD layer acted as a charge-trapping layer. On the basis of the above results, a schematic diagram of the energy band under zero-bias for the device is presented in Figure 8a. When *V*_SET1_ was applied to the ITO electrode, the device switches from HRS to IRS, as electrons were initially trapped in the GO@PBD layer, as shown in Figure 8b. However, the electrons were only captured in the first GO@PBD layer, because the relatively large energy barrier between the GO@PBD layer and the PMMA layer prevented the generation of a sufficient internal field to form conducting filaments. When the applied voltage increased to *V*_SET2_, the external electric field was large enough for the electrons to overcome the barrier from the trap level to the LUMO level of the PMMA layer and to be trapped by the second nanocomposite film through an SCLC mechanism, as shown in Figure 8c. Therefore, the almost complete occupation of the second GO@PBD nanocomposite layer by the electrons was sufficient to build an internal field high enough to form conducting filaments in the stacking layer, which resulted in an Ohmic current in LRS, with the device being switched from IRS to LRS. In contrast, when *V*_RERET_ was applied to the device, the current started to decrease as a result of the emission of the electrons from the nanocomposite film, which destroyed the conducting paths. The electrons trapped in the first GO@PBD nanocomposite layer at a particular applied bias were ejected back to the ITO electrode, as shown in Figure 8d. When the voltage continued increasing, the conducting channel was not completely destroyed, and the device changed from IRS to HRS, as shown in Figure 8e. 

## 4. Conclusions

In conclusion, we successfully demonstrated a nonvolatile organic memory using a small molecule noncovalently bonded to graphene oxide (GO@PBD) as an active layer sandwiched between ITO bottom and Ni top electrodes. This is the first successful attempt to fabricate an organic nonvolatile memory device using graphene-based small molecules with a cheap spin-coating technique. A heterostructure consisting of an insulating polymer PMMA sandwiched between double GO embedded in PBD nanocomposite layers was described. More inspiringly, a programmable multi-bit memory system was achieved under sweeping voltages, which can significantly enhance the storage density. An innovative architecture for multi-bit memory applications was provided, which has great potential for further developments.

## Figures and Tables

**Table 1:** TGA results for PBD, GO@PBD nanocomposite, and GO.

Materials	T_0.1_ (°C)	*T*_0.5_ (°C)
PBD	345	399
GO@PBD nanocomposite	236	388
GO	87	-

**Table 2:** Optical properties of PBD, GO@PBD, GO, and PMMA in solution.

Materials	UV *λ*_abs_ (nm)	UV *λ*_edge_ (nm)	*E*_g_ (eV)
PBD	322	363	3.375
GO@PBD	340	374	3.324
GO	243	300	4.143
PMMA	231	293	4.242
